# Impacts of extreme agroclimatic indicators on the performance of coffee (*Coffea arabica* L.) aboveground biomass in Jimma Zone, Ethiopia

**DOI:** 10.1016/j.heliyon.2022.e10136

**Published:** 2022-08-08

**Authors:** Fedhasa Benti Chalchissa, Girma Mamo Diga, Gudina Legese Feyisa, Alemayehu Regassa Tolossa

**Affiliations:** a Jimma University, Department of Natural Resource Management, Jimma, Ethiopia; b Ethiopia Agricultural Research Institute, Addis Ababa, Ethiopia; c Addis Ababa University, Center for Environmental Science, Addis Ababa, Ethiopia

**Keywords:** Agroclimatic indicator, Bove ground biomass, Artificial neural network model, Coffee tree, Microclimates

## Abstract

Estimating crop biomass is critical for countries whose primary source of income is agriculture. It is a valuable indicator for evaluating crop yields and provides information to growers and managers for developing climate change adaptation strategies. The objective of the study was to model the impacts of agroclimatic indicators on the performance of aboveground biomass (AGB) in Arabica coffee trees, a critical income source for millions of Ethiopians. One hundred thirty-five coffee tree stump diameters were measured at 40 cm above ground level. The historical (1998–2010) and future (2041–2070) agroclimatic data were downloaded from the European Copernicus climate change services website. All datasets were tested for missing data, outliers, and multicollinearity and were grouped into three clusters using the K-mean clustering method. The parameter estimates (coefficients of regression) were analyzed using a generalized regression model. The performance of coffee trees' AGB in each cluster was estimated using an artificial neural network model. The future expected change in AGB of coffee trees was compared using a paired t-test. The regression model’s results reveal that the sensitivity of *C. arabica* to agroclimatic variables significantly differs based on the kind of indicator, RCP scenario, and microclimate. Under the current climatic conditions, the rise of the coldest minimum (TNn) and warmest (TXx) temperatures raises the AGB of the coffee tree, but the rise of the warmest minimum (TNx) and coldest maximum (TXn) temperatures decreased it (P < 0.05). Under the RCP4.5, the rise of consecutively dry days (CDD) and TNx would increase the AGB of the coffee tree, while TNx and TXx would decrease it (P < 0.05). Except for TXx, all indicators would significantly reduce the AGB of coffee trees under RCP8.5 (P < 0.05). The average values of AGB under the current, RCP4.5, and RCP85 climate change scenarios, respectively, were 26.66, 28.79, and 24.41 kg/tree. The predicted values of AGB under RCP4.5 and RCP8.5 will be higher in the first and third clusters and lower in the second cluster in the 2060s compared to the current climatic conditions. As a result, early warning systems and adaptive strategies will be necessary to reduce the detrimental consequences of climate change. More research into the effects of other climatic conditions on crops, such as physiologically effective degree days, cold, hot, and rainy periods, is also required.

## 1 Introduction

Estimating crop biomass is critical for countries whose primary source of income is agriculture. The dry biomass of the crops is directly linked to agricultural production and is used to predict crop yields (Geng et al., 2021; Reisi-Gahrouei et al., 2019). It is an essential indicator for evaluating crop yields and provides better information to growers and managers for developing climate change adaptation strategies such as efficient fertilizer applications (Byrareddy et al., 2019), irrigation requirement determination (Byrareddy et al., 2020; Guo et al., 2010) disease and weed control strategies (Castex et al., 2018; González García et al., 2015; Juroszek and Von Tiedemann, 2011) and warning decision-makers about the possibility of crop yield shortages to improve productivity (Wang et al., 2021).

The biggest challenge currently facing agricultural producers worldwide is climate change. Extreme climatic index occurrences have become more frequent and more severe in recent decades due to climate change and global warming (Ramalho et al., 2014; Schroth et al., 2016). The frequency and intensity of extreme events have increased significantly in recent decades due to climate change and global warming (Perkins et al., 2012). Extreme weather events (droughts, floods, heatwaves, colder and warmer temperatures, changes in precipitation patterns), a decline in agricultural production, and increased incidence of pests and diseases have all been exacerbated across the broads by climate change (Bernstein et al., 2007; Schroth et al., 2016; WMO, 2010). Agriculture production is based on climate and meteorological conditions. Today, changes in temperature and precipitation directly affect agricultural production (Chemura et al., 2015). Changing rainfall patterns and rising temperatures may complicate agricultural development, shorten growing seasons and increase pest and disease distributions. Maize rice, wheat sorghum, pulses, oilseeds (Casas, 2017), coffee, and cocoa (Schroth et al., 2016; Sousa et al., 2019) are among the most vulnerable crops to climate change and variability.

*C. arabica* is one of Ethiopia’s most valued commodities, but its products are impacted by climate change (Bento et al., 2010; Filipe et al., 2015; Hirons et al., 2018; Rahn et al., 2018). Studies on arabica plants' responses to climate stresses like extreme cold, heat, and drought show that coffee yield and quality decline in less-than-ideal growing conditions (Muslihah et al., 2020; Ramalho et al., 2014). The Arabica beans can’t grow big enough outside of their ideal temperature. According to the IPCC (2018), rising temperatures, more frequent and severe droughts, and more seasonal changes in the Bean Belt could be dangers for Arabica coffee.

*C. arabica* is very sensitive to climatic factors, and it cannot withstand low temperatures, nor does it tolerate intense heat either (Wang et al., 2008; Chalchissa et al., 2022). As a result, it is widely cultivated on relatively chilly mountain slopes in the tropics (Moat et al., 2017). Warming temperatures severely affect the growth and development of *C. arabica* (Damatta and Ramalho, 2006). The amount of photosynthetic active radiation (PAR) collected by the crop during its life cycle is related to crop biomass buildup (Monteith, 1972). Of the 50% of photosynthetically active radiation (0.4μ–0.7μm), only 2–10% of this supports photosynthesis. The plant’s leaves absorb blue and red wavelengths of visible solar radiation (Buckner et al., 2016). Warmer temperatures hinder the economic exploitation of the coffee plant by disrupting metabolic and absorption processes in shoots and leaves, resulting in imitative plant growth performance (Sanquetta et al., 2011). It hastens evaporation, decreases soil moisture, and halts CO2 fertilization and photosynthetic activity (Vieira et al., 2018).

On the other hand, the colder temperatures hinder water and nutrient absorption and transport inside the plant, decreasing development and growth and, eventually, total plant biomass (Damatta et al., 2018). Droughts in the tropics are aggravated by excessive sun radiation and temperatures, resulting in multidimensional stress on coffee plants, and these concerns are predicted to become increasingly prevalent in coffee-growing locations (Damatta and Ramalho, 2006). In general, climate change alters how plants interact with their surroundings, which impacts the biomass accumulation of coffee plants (Prieto et al., 2009; van Oijen et al., 2010).

Ethiopia is the largest coffee producer in Africa, but its primary coffee-growing locations will likely no longer be influenced by cultivating this commodity due to excessive temperature and precipitation indices (Davis et al., 2012b; Moat et al., 2017a, 2017b). Meanwhile, increasing temperatures may endanger native coffee trees, a vital repository of coffee’s primordial genetic diversity that grows wild in Ethiopian forests. Many researchers have been working tirelessly to understand how climate suitability influences coffee plants and devise practical solutions to mitigate the severity of the consequences (Bunn et al., 2015; Davis et al., 2012b; Godfrey et al., 2018; Muslihah et al., 2020; Wagner et al., 2021). However, none of them have looked at *C. arabica’s* physiological response to agroclimatic variables, so no data on biomass production variation has been published. The current study aimed to make a difference by assessing extreme agroclimatic impacts on the *C. arabica* trees' aboveground biomass under medium and higher emission scenarios using ground and satellite data in response to the existing research gaps.

## 2 Methodology

### 2.1 A description of the study area

**Figure 1:**
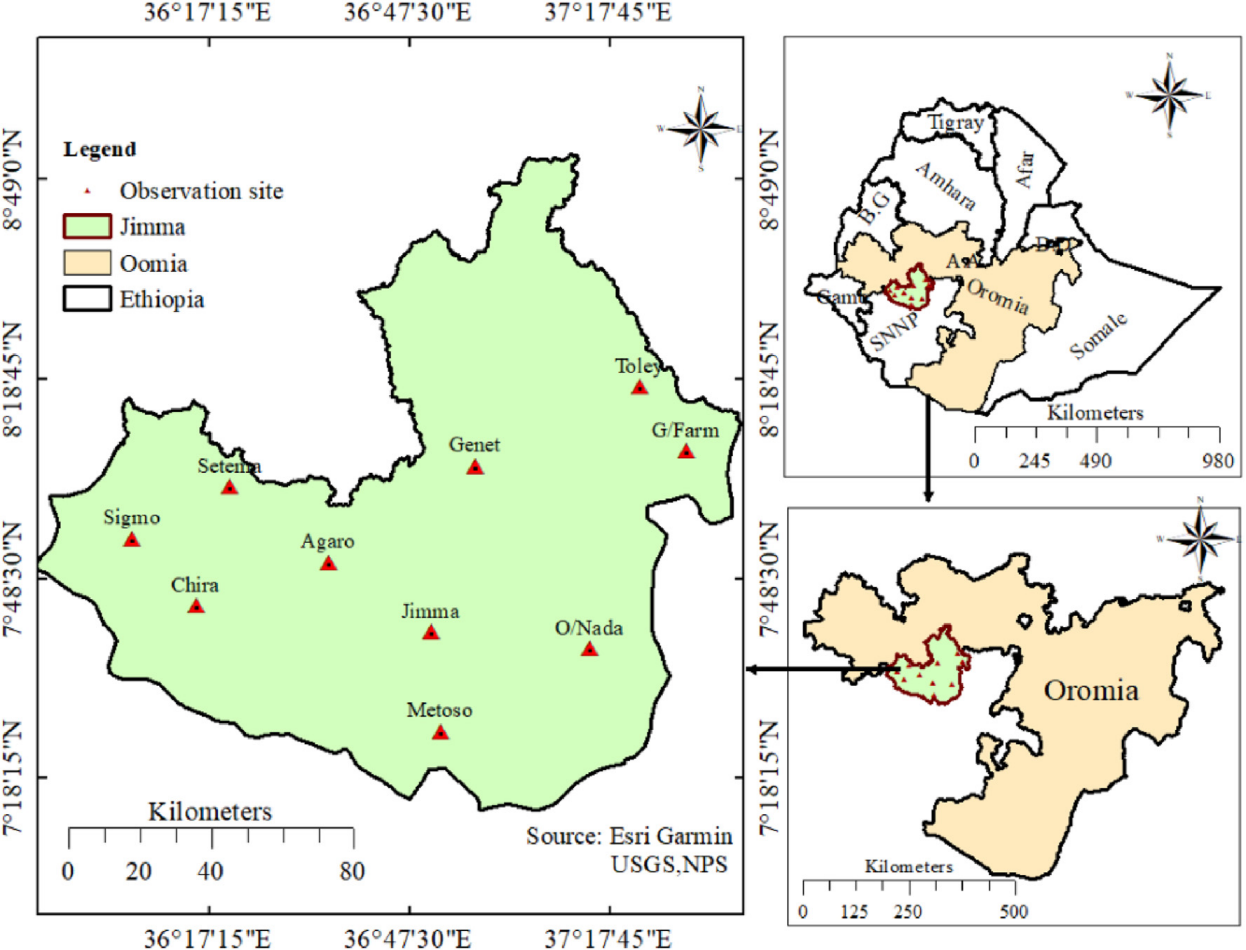
Map of the research area with the locations of meteorological stations.

The study was conducted in the Jimma zone of Oromia National Regional State, Ethiopia. as shown in Figure 1, Jimma is located 357 km to the southwest of Addis Abeba, between 7°13′ and 8°56′ N and 35°49′ and 38°38′ E. The Jimma Zone consists of various landforms with an altitude of 871 m–3231 m above sea level. It is characterized by a tropical highland climate with high rainfall, warm temperatures, and low humidity. Its average annual temperature is between 11 and 25, and the total annual precipitation is between 1200 and 2400 mm, and the rainy season often lasts from February to October (Eshetu, 2018; Gemeda et al., 2020). Similar to southern and south-eastern Ethiopia, spring and summer rains in southwestern Ethiopia have decreased by 15–20% since 1970 (Sisay, 2018). The study area has three major climatic zones: subtropical, temperate, and tropical or warm areas, which account for 78%, 12%, and 10%, respectively (Diro and Erko, 2019). *C. arabica* is one of the most important cash crops grown in the southwestern regions because of its origin and climate suitability for this the environment (Davis et al., 2012a; Moat et al., 2017a, 2017b).

### 2.2 Data collection

#### 2.2.1 Biomass data

The preferential sampling technique was adopted since it assures that all of the necessary samples have a chance of being involved in the research. The three types of coffee sub-zones defined by Benti et al. (2022) were used to sample coffee trees in the research area. Three small-scale representative coffee farms (50 m × 60 m) were randomly selected from sub-zones. Each farm was then divided into five plots (20 m × 30 m), with three coffee trees from each plot were selected to measure diameters. A total of 135 coffee trees were chosen at 40 cm (D_40_ cm) above ground level using the method of Negash et al. (2013).

A non-destructive technique for aboveground biomass (AGB) estimation was used because property rights regulations may not allow cutting coffee without special authorization. The samples of stand coffee trees with a diameter greater than or equal to 2 cm at 40 cm aboveground level were measured using a tap meter as described in the method (Negash et al., 2013). Samples of 51, 45, and 42 coffee trees from the 1st, 2nd, and 3rd sub-Zones or a total of 135 coffee trees' stump diameters, were measured at 40 cm (D_40_ cm) above ground level. Sample trees' locations were recorded during data collection.

#### 2.2.2 Agroclimatic indicator data

The European Copernicus Climate Change Services (C3S) provided historical agroclimatic indicator data from 1981 to 2010 and future projections from 2041 to 2070. The datasets are freely available in a NetCDF format file on the website: https://climate.copernicus.eu/. Every agroclimatic indicator is derived from daily precipitation and minimum, mean, and maximum temperatures. The WFDEI (Watch Forcing Data methodology applied to ERA-Interim) was used to reanalyze agroclimatic indicators for historical and future time periods. This product offers bias-corrected climate data from five CMIP5 General Circulation Models (MIROC-ESM-CHEM, IPSL-CM5A-LR, NorESM1-M Mode, GFDL-ESM2M, HadGEM2-ES). This dataset contains indices with a spatial resolution of 0.5 ° × 0.5 ° on a latitude and longitude grid. These climate datasets are provided by the Inter-Sectoral Impact Model Intercomparison Project (ISIMIP2). ISIMIP is a community-driven climate impact modeling initiative that aims to contribute to a quantitative and cross-sectoral synthesis of climate change’s different impacts (ECMWF, 2019). The medium (RCP4.5) and higher (RCP8.5) emission scenarios were considered for the current study.

#### 2.2.3 Data screening and clustering

**Figure 2:**
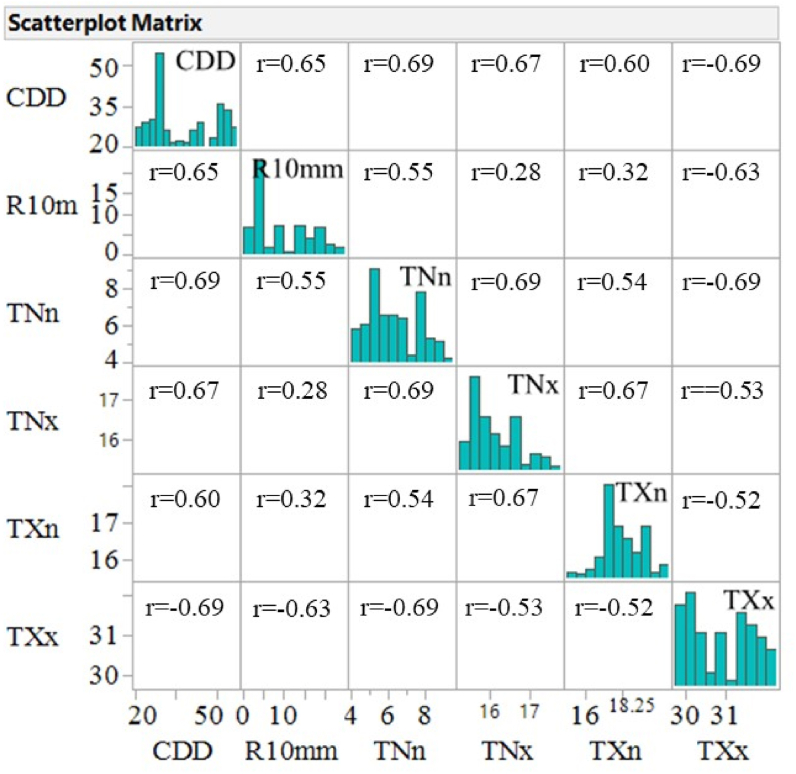
Correlation Matrix of least correlated agroclimatic indicators employed for this study.

Data must be screened before further statistical analysis to ensure that it is useable, reliable, and valid for testing causal theories. Missing values and outliers are the two factors that increase the degree of bias, degrade the efficiency of the data, and reduce the reliability, validity, and generalizability of study results (Kwak and Kim, 2017). The two components were investigated using a machine learning method (utilizing JMS software) to handle data with missing values and outliers, and fortunately, our datasets had no missing values or outliers. It might be because the datasets were bias-corrected at the start (ECMWF, 2019). Multicollinearity reduces the statistical strength of the regression model by reducing the precision of estimated coefficients. It affects only the specific independent variables that are correlated (Wonsuk et al., 2014). The coefficients become particularly sensitive to small changes in the variables. A Pearson correlation coefficient was also employed to identify highly correlated agroclimatic variables. One of the two highly correlated agroclimatic variables was removed when the absolute values of the correlation coefficient between the variables were |r| > 0.7. Because multicollinearity makes the model’s output unreliable (Dormann et al., 2013). Figure 2 shows the selected agroclimatic variables with a low correlation coefficient, or |r|, less than 0.70 and their normal distributions.

**Figure 3:**
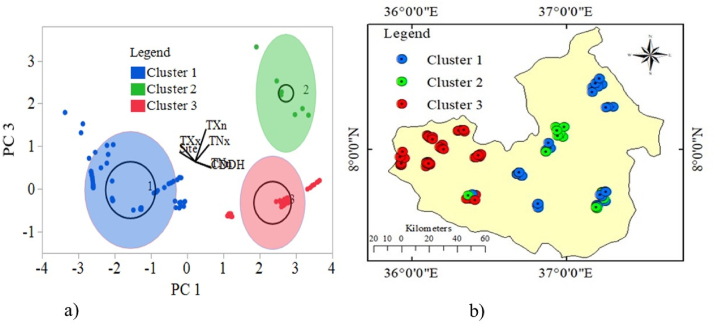
Input data as clustered into three Gaussian distributions.

A data clustering technique was used to deal with this multivariate input data. This process may be used to evaluate the geographical variation of input data (SAS, 2020). The total sample location of coffee trees was reduced from 135 to 127 by removing eight sample coffee tree locations with equivalent agroclimatic datasets for the dependent variables (Table 1). The observed AGB of the coffee trees and extreme climate input data were categorized into three clusters based on their mean values using the K-Means clustering technique. The clustering technique produces groups of items in the same category that have comparable features (Morissette and Chartier, 2013). In this scenario, K-mean clustering assigns every data point to the nearest centroid, which means K different randomly-initiated points in the data. After all the data points have been assigned, the centroid is moved to an average of them (Figure 3a). The data was redistributed and mapped with ArcGIS as shown in Figure 3b.

**Table 1:** Measured AGB *C. arabica* tree and selected agroclimatic indicators.

Code	Description	Unit
40cm	Diameter coffee tree at 40 cm above ground surface	cm
AGB	Aboveground biomass of C, arabica tree	kg
CDD	Annual maximum number of consecutive dry days when daily rainfall <1 mm	Day
R10mm	Annual heavy precipitation days when daily total precipitation ≥10 mm day	Day
TNx	Annual maximum values of minimum temperature when daily minimum temperature ≥15 °C	°C
TNn	Annual minimum values of minimum temperature when daily minimum temperature ≤10 °C	°C
TXn	Annual minimum values of Maximum temperature when daily maximum temperature ≤20 °C	°C
TXx	Annual maximum value of maximum temperature when daily maximum temperature >25 °C	°C

Table 2 represents the statistical summary of the observed *C. arabica’s* AGB and historical agroclimatic indicators between 1981 and 2010. The agroclimatic datasets were converted to a comma-separated value (CSV) file or geospatial data to point data using ArcGIS (Phillips, 2017). It might aid in obtaining the exact agroclimatic indicators at the sites of each sample coffee tree. The outliers and missing data were screened using JMP software. A partial correlation analysis was carried out to choose the agroclimatic indicator having a higher relationship with the AGB of the *C. arabica* trees. The agroclimatic extremes at its normal distribution and partial correlation coefficient of more than 0.25 and less than -0.25 to the *C. arabica’s* AGB were chosen according to Bühlmann et al. (2010).

**Table 2:** Statistical summary of *C. arabica* growth performance and current agroclimatic indicators' data.

Cluster	Parameter	N	D_40_cm	AGB_*O*_	CDD	R10P	TNx	TNn	TXn	TXx
1	Mean	27	15.17	33.93	26.59	5.65	5.22	15.9	16.65	31.74
	SD	27	0.84	3.62	3.53	0.02	0.71	0.21	0.49	0.15
	CV	27	5.54	10.67	13.28	0.35	13.60	1.32	2.94	0.47
2	Mean	49	14.25	29.94	32.87	9.61	5.71	15.92	16.78	30.78
	SD	49	0.86	3.55	5.56	8.08	0.38	0.36	0.36	0.4
	CV	49	6.04	11.86	16.92	84.08	6.65	2.26	2.15	1.30
3	Mean	51	11.41	19.52	52.79	15.49	7.82	16.77	17.35	29.93
	SD	51	1.65	5.3	4.63	4.08	0.76	0.44	0.56	0.19
	CV	51	14.46	27.15	8.77	26.34	9.72	2.62	3.23	0.63
Overall	Mean	127	13.47	26.66	37.42	10.25	6.25	16.2	16.93	30.82
	SD	127	2.02	7.37	11.98	6.84	1.29	0.56	0.56	0.74
	CV	127	15.00	27.64	32.01	66.73	20.64	3.46	3.31	2.40

#### 2.2.4 Above ground biomass estimation

A species-specific equation was utilized to calculate the AGB of *C. arabica* trees, and the average AGB per thermal climatic zone was recorded separately. The aboveground biomass data was calculated using the allometric equation (Eq. (1)) established by Negash et al., (2013), for a specific coffee arabica species in the Gedeo zone, closer to the study area.

(1) $$AGB_{Coffee}=0.147{\left(D_{40cm}\right)}^{2}$$

where AGB indicates above-ground biomass of coffee trees, D_40_ cm indicates stump diameter at 40 cm above ground level.

According to Negash et al. (2013), the square power equation has the best rate of goodness-of-fit (R-squared = 80%) for estimating the total and component biomass of arabica coffee tree.

### 2.3 Modeling above-ground biomass under climate change

#### 2.3.1 Model validation and testing

Different model comparisons were carried out before constructing predictive models to choose the best fit model for the dataset. Validation is a technique of estimating model parameters with part of a data set and assessing model predictive abilities with the remaining data set (SAS, 2020). It can lessen the threat of model overfitting when dealing with complex data. Both AGB and agroclimatic indicators' datasets were split into three of the validation columns: training 70%, validation 15%, and testing 15%. The training dataset was employed to assess model parameters. The validation dataset was used to select a model with higher accuracy for predicting the outcome. After a model was selected, the testing dataset was used toevaluate the predictive ability of the model.

he coefficient of determination (R-square), root mean square error (RMSE), and mean absolute error (MAE) values for training, validation, and tests were used to evaluate and compare the model performance (SAS, 2020).

R-square is known as the coefficient of determination. It aids in describing the strength of a relationship between a dependent variable (the AGB of *C. arabica*) and independent variables (climate extremes). It shows how close the actual data values are to the regression line. The R-squared value ranges from 0 to 1, with 0 indicating that the model does not match the given data and 1 indicating that the model fits the dataset correctly. An adjusted R-squared greater than 0.75 is very good for demonstrating accuracy. A model assessment helped this work investigate the impacts of local climatic change on *C. arabica’s* AGB performances. Eq. (2) was used to calculate the R-squared.

(2) $$R^{2}=1-\frac{RSS}{TSS}$$

where R2 is the coefficient of determination, RSS is the sum of squares of residuals, and TSS is the total sum of squares.

A Root Mean Square Error (RMSE) is the standard deviation of the errors that occur while predicting a dataset. It is similar to the Mean Squared Error (MSE), except that the root of the integer is used when calculating the model’s accuracy (SAS, 2020). It tells us how concentrated the data is around the line of best fit. The lower values of RMSE indicate a better fit of the model to the dataset. It was calculated by Eq. (3).

(3) $$RMSE=\sqrt{\frac{\sum_{i=1}^{n}\left(\hat{y_{i}}-Y_{i}\right)}{n}}\;$$

where RMSE is the root average square error, y is a predicted AGB, Y is the actual AGB, and i = 1, 2, 3, ...127, and n is the number of the sample population.

The absolute mean error measures the average magnitude of the error without considering direction. The high values of MAE are undesirable, and the low ones indicate the better accuracy of the model to fit the datasets. Eq. (4) was utilized to calculate absolute mean error.

(4) $$\text{MAE ​}=\text{ ​}\frac{1}{n}\overset{n}{\underset{i=1}{\sum }}\left|Y_{i}-\hat{y_{i}}\right|$$

where MAE stands for the absolute difference between the actual values of AGB and the projected values of AGB, Y is measured AGB, and $\hat{y_{i}\;}$ is predicted AGB, i = 1, 2, 3, ...127. If the result has a negative sign, it is ignored in absolute terms.

#### 2.3.2 Model development

**Figure 4:**
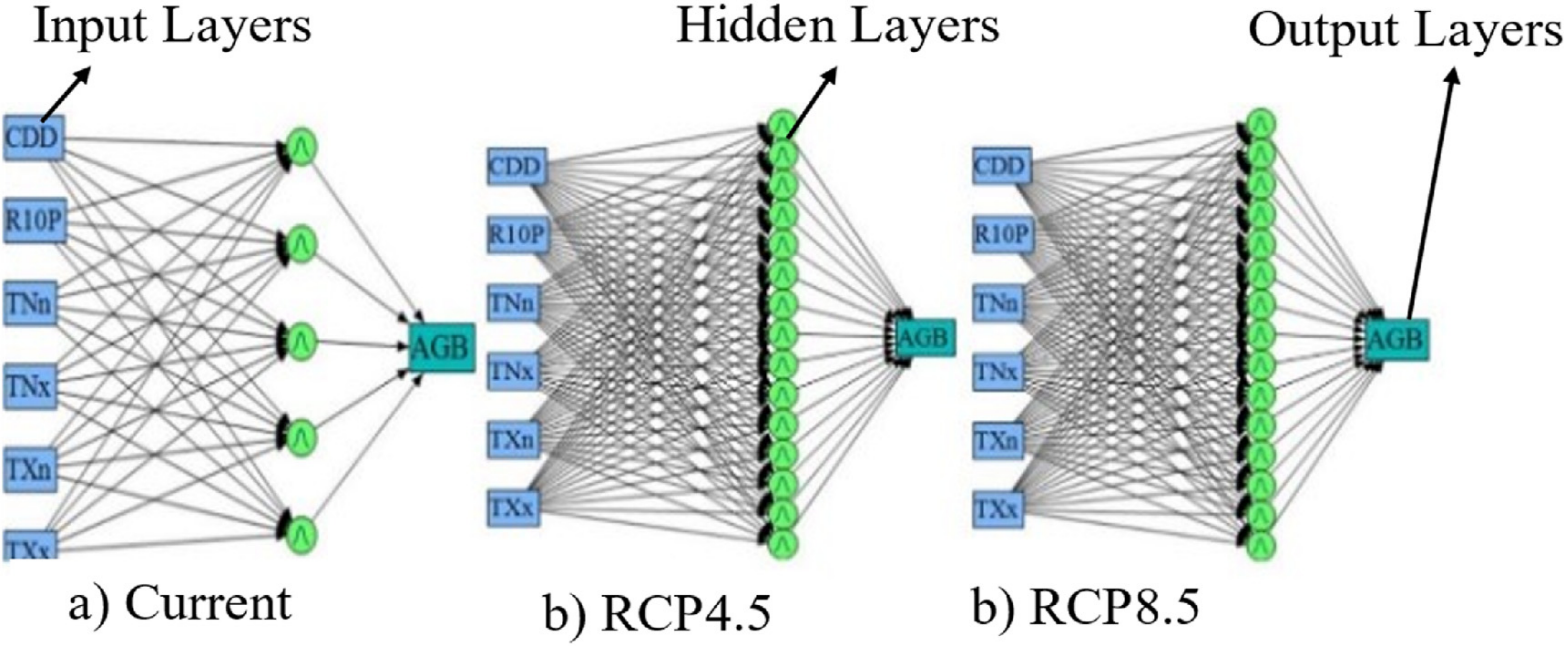
Structure of an Artificial Neural Network model with 5, 15, and 15 neurons for present, RCP4.5, and RCP8.5 climate change scenarios.

Figure 4 depicts a diagram of the artificial neural network (ANN) model for current and future climate change scenarios. A neural network, which has the best predictive model that fits AGB and agroclimatic indicators data, was chosen to assess the impacts of climate change on the AGB performance of *C. arabica*. A neural network is often known as an artificial neural network (ANN). It is a mathematical model that represents the system of biological brain networks. This model describes the relationship between the predictors and the response variables very well (SAS, 2020). It recognizes underlying relationships in datasets through a process that acts like the human brain (SAS, 2020). Therefore, we created the artificial neural network models for the historical and future 2060s under the RCP4.5 and RCP8.5 climate change scenarios using the commercial statistical program JMP version 14 (SAS, 2020). The model consists of three layers of neurons, as illustrated in Figure 4; the input layer, the hidden layer, and the output layer. As part of the operation with this type of neural network, a network of coffee trees in AGB was generated (Wu et al., 2019). Both AGB and agroclimatic indicators' datasets were submitted into input layers, were analyzed to produce weighted values, and these weighted values were then transmitted to the series of hidden layer nodes that provide predicted AGB values and then, transfered them into the output layer (Figure 4). The estimated output of AGB was calculated in the output layers using Eq. (5) as described by Wu et al. (2019).

(5) $$A\hat{G}B=\text{f ​}\left(\sum {\text{W}}_{\text{i}}{\text{X}}_{\text{i}}-\theta \right)$$

where AGB is aboveground biomass, f is the activation function. W is the weight of dry biomass, X is agroclimatic indicators, and θ is the threshold.

#### 2.3.3 Model evaluation

Table 3 depicts a trial-and-error method of artificial neural network model’s performance evaluation. During the model building process, the performance of the models varied widely. Therefore, many neurons in a hidden layer were chosen using a trial-and-error technique until a good result was achieved. Because artificial neural networks model with too many neurons and hidden layers memorize the input data, those with few neurons and hidden layers may not produce accurate predictions (Danso-Amoako and Prasad, 2014). Increasing the number of neurons sometimes increases the values of the R-square while reducing the RMSE (Domingues et al., 2020). As a result, five feedforwards were created for each TanH, linear and Gaussian activation functions using AGB, and historical and future agroclimatic indicators. Finally, the Gaussian activation function with the maximized R-square and minimized RSME and MAE was optimized at 5, 15, and 15 neurons for current, RCP4.5, and RCP8.5, respectively (Table 3).

**Table 3:** Trial and Error method of Artificial neural network model’s performance evaluation.

Scenarios	Number of Neuron	Training			Testing		
		R-squared	RMSE	MAE	R-squared	RMSE	MAE
Current	**5**	**0.89**	**2.42**	**1.75**	**0.92**	**2.04**	**1.61**
	10	0.89	2.45	1.80	0.92	2.04	1.63
	15	0.89	2.49	1.86	0.91	2.13	1.71
	20	0.89	2.49	1.84	0.90	2.19	1.74
	25	0.88	2.54	1.99	0.90	2.21	1.77
RCP4.5	5	0.91	2.08	1.67	0.88	2.38	1.86
	10	0.97	1.23	0.91	0.83	2.68	2.18
	**15**	**0.99**	**0.70**	**0.48**	**0.94**	**1.71**	**1.35**
	20	0.99	0.75	0.59	0.87	2.81	2.32
	25	0.97	1.36	1.10	0.84	2.36	1.70
RCP8.5	5	0.912	2.12	1.71	0.89	2.44	1.83
	10	0.925	2.02	1.58	0.89	2.39	1.71
	**15**	**0.94**	**1.80**	**1.37**	**0.92**	**2.008**	**1.37**
	20	0.94	1.74	1.32	0.89	2.29	1.60
	25	0.94	1.77	1.34	0.91	2.20	1.54

Note: The Bolded number indicated the best performance of ANN model at 5, and 15 neurons.

### 2.4 Model parameter estimates

A change in a predictor variable (agroclimatic indicator) was associated with a change in a response one (*C. arabica’s* AGB) while all other predictors remain constant. It’s critical to analyze whether agroclimatic indicators positively or negatively influence the *C. arabica* AGB’s accumulation performance. These unknown parameter estimates were analyzed using a Generalized Regression model (GRM). This model generates multiple regression weights, Wald Chi square, and reduces the multicollinearity of the predictors. Multicollinearity is defined as the variance of inflation factors (VIP) (Derringer and Suich, 1980; SAS, 2020). The squared ratio of the estimate to the standard error of the corresponding predictor is the Wald Chi-Square test statistics. The probability that a particular Wald Chi2 test statistic is the same as or more extreme than the significant level (α = 0.05) is known as Pr > Chi2 (Indumathi et al., 2014).

### 2.5 Estimation of aboveground biomass change under climate scenarios

The statistics of the batch values; the mean, standard deviations (SD), standard error (SE), and coefficient of variation (CV) of the observed and predicted aboveground biomass of the coffee trees under different ecoclimatic clusters and climate change scenarios were analyzed using JMP software. The mean variation between the predicted AGB under current and future climate change scenarios was determined using a paired T-test. The relative difference between the current and the future predicted AGB in the current and in the 2060s was calculated using Eq. (6).

(6) $$RA\hat{G}B=\left(\frac{A\hat{G}B_{f}-A\hat{G}B_{C}}{A\hat{G}B_{C}}\right)\times 100$$

where $RA\hat{G}B$ is the relative change of above-ground biomass, $A\hat{G}B_{f}\;$ is future above-ground biomass, and $A\hat{G}B_{C}\;$ is the predicted current above-ground biomass of arabica coffee trees.

## 3 Results

### 3.1 Model validation and testing

The results of the model comparison analysis are illustrated in Table 4. According to the validation study, an artificial neural network (ANN) model fits the dataset best, followed by a general regression model. The ANN model is more accurate than generalized regression and bootstrap forest models, with the highest value of R-squared, the lowest values of RMSE, and the highest MAE. The validation set’s R-Square scores for ANN model were 0.89, 0.96, and 0.94, indicating that the model is superior to general regression and Bootstrap to fit the data that was not used to train it. This finding coincides with Salmayenti et al.’s (2017) prediction of the relationship between rainfall variability and the El Nino Southern Oscillation and the Indian Ocean Dipole (IOD) in Indonesia.

**Table 4:** Models' validation as three distinct models compared for data goodness of fit (n = 32).

Scenarios	Predictive Models	R^2^	RMSE	MAE
Current	**Artificial neural network**	**0.89**	**0.11**	**0.97**
	Generalized regression	0.79	0.21	1.65
	Bootstrap forest	0.42	0.58	2.49
RCP4.5	**Artificial neural network**	**0.96**	**0.08**	**1.22.**
	Generalized regression	0.79	0.21	1.65
RCP48.5	Bootstrap forest	0.42	0.58	2.49
	**Artificial neural network**	**0.94**	**1.36**	**2.24**
	Generalized regression	0.79	0.21	1.65
	Bootstrap forest	0.42	0.58	2.49

Note: RASE and MAE indicate root average square error and mean absolute average error, respectively.

### 3.2 Evaluation of model performance

**Figure 5:**
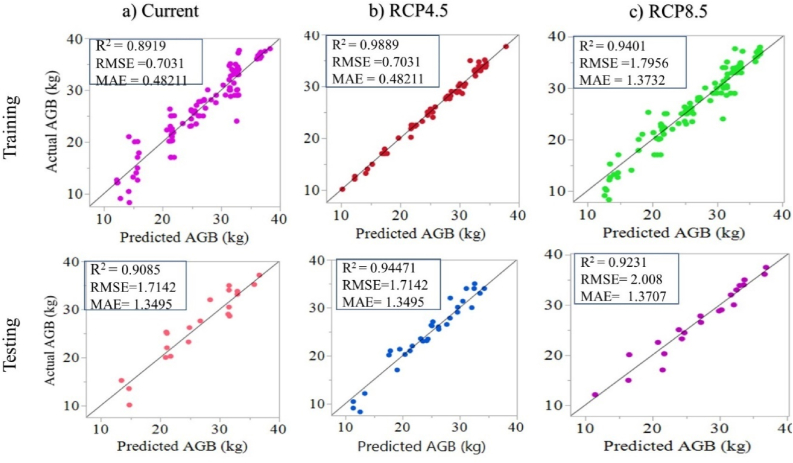
Evaluation of the ANN Model's performance under current, RCP4.5, and RCP8.5 climate conditions.

Figure 5 displays the R-square and RMSE values produced by the Gaussian activation functions when comparing actual and predicted AGB of *C. arabica*. The R-square (R^2^) values, RMSE, and MAE at the top of the plots show a significant association between actual and predicted AGB in training and testing. The 5th number of neurons in the hidden layer produced the highest R-square values and the lowest RMSE and MAE values in the current climate conditions. The higher R-squared and the lower RMSE and MAE values were found on the 15th number of neurons for the RCP4.5 and RCP8.5 climate scenarios. The highest R-square value combined with the lowest RMSE and MAE values in data testing indicates the model is performing effectively (Domingues et al., 2020; SAS, 2020).

### 3.3 Model parameter estimates under current and future cimate change scenarios

#### 3.3.1 Current (1981–2010)

Table 5 shows the standardized regressive weight (coefficient) of agroclimatic parameters related to the AGB of coffee trees. The results of the generalized regression model indicate that the AGB of coffee trees has an asymmetric relationship with acute agroclimatic indicators, given that changes in agroclimatic variables cause changes in AGB at a significant level of P < 0.05. Under the current conditions, every unit increase in CDD, TNx, and TXn significantly decreased the AGB of the C. arabica tree, while the increases in R10mm, TNn, and TXx improved AGB (P < 0.05). It implies that when the annual mean values of CDD, TNx, and TXn increase by one unit, the mean values of AGB wereestimated to declined by 1.11, 2.72, and 1.56 kg/tree, respectively. Nonetheless, when the mean values of R10mm, TNn, and TXx increase by one unit, the corresponding mean values of AGB were estimated to be significantly raised by 0.36, 7.48, and 2.86 kg/tree, respectively.

**Table 5:** Standardized regressive weight of agroclimatic indicators associated with coffee trees' AGB.

Agroclimatic predictors	scenarios	Estimate	Std Error	Wald ChiSquare	Prob > ChiSquare	VIF
CDD	Current	−1.11	0.14	67.20	<.0001	2
	RCP4.5	0.25	0.02	148.86	<.0001	1
	RCP8.5	−0.10	0.02	21.52	<.0001	3
R10mm	Current	0.36	0.07	29.46	<.0001	6
	RCP4.5	0.03	0.02	1.93	0.17	5
	RCP8.5	−0.05	0.02	6.23	0.01	1
TNn	Current	7.48	1.16	41.83	<.0001	11
	RCP4.5	0.47	0.46	1.03	0.31	2
	RCP8.5	−1.36	0.51	6.99	0.01	2
TNx	Current	−2.72	1.26	4.66	0.03	10
	RCP4.5	5.65	1.07	28.12	<.0001	6
	RCP8.5	−4.93	1.12	19.50	<.0001	6
TXn	Current	−1.56	0.79	3.90	0.05	5
	RCP4.5	−3.36	1.17	8.26	0.001	7
	RCP8.5	2.35	1.29	3.31	0.07	8
TXx	Current	2.86	0.45	40.67	<.0001	3
	RCP4.5	−1.05	0.43	5.87	0.02	2
	RCP8.5	3.02	0.47	41.45	<.0001	2

Note; VIF indicates the Impacts of Variance Inflation factors on the models between 1 and 10 variation.

#### 3.3.2 Future (2041–2070)

The result indicates that *C. arabica’s* growth performance (AGB) was supposed to be asymmetrically correlated to agroclimatic factors in the 2060s. Increases in the mean values of CDD and TNx are expected to have a significant positive effect on the performance of the *C. arabica tree’s* AGB (P < 0.0001) when the remaining indicators in the RCP4.5 scenarios are kept constant (Table 1). It implies that for every one-day increase in CDD and one degree Celsius in TNx, the AGB levels rise by 0.25 and 5.56 kg, respectively, assuming all other variables stay constant. A one-degree Celsius increase in the annual mean values of TXn and TXx in the RCP4.5 scenario will result in AGB losses of 3.36 and 1.05 kg per tree, respectively (P < 0.05).

Nevertheless, it was anticipated that the impacts of agroclimatic indicators on the AGB performance of C. arabica trees under the RCP8.5 climate change scenarios would be different from or contradict those on AGB under the RCP4.5 climate change scenarios. Under the RCP8.5, the AGB of *C. arabica* was anticipated to decrease for every 1 unit rise in the annual mean of CDD, R10mm, TNn, and TNx. It means that the AGB of the crop plant will significantly decrease by 0.10, 0.5, 1.35, and 4.5 kg for every unit increase of CDD, R10mm, TNn, and TNx, respectively (P < 0.01). Unlike the RCP4.5, the RCP8.5 forecasts an annual increase of one degree Celsius in TXn and TXx will that considerably increase AGB (P < 0.0001) by 2.35 and 3.02, respectively.

#### 3.3.3 Interactive matrix of agroclimatic indicators

**Figure 6:**
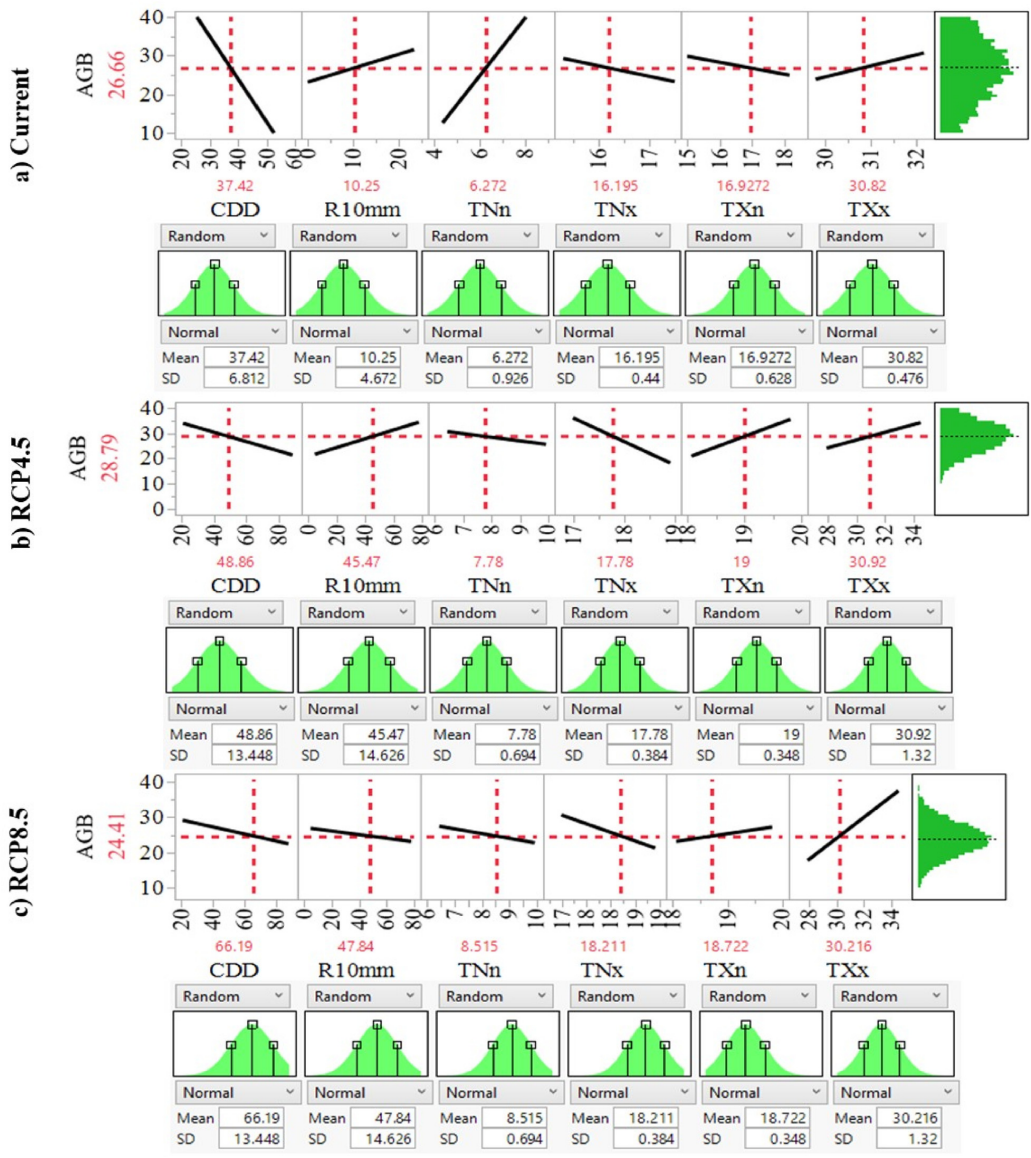
Predictive profilers demonstrate for agroclimatic factors settings at the normal distribution of coffee tree’s AGB under Current (a), RCP45 (b) and RCP8.5 (c) climate change scenarios.

Figure 6 demonstrates the general regression analysis between the AGB of the *C. arabica* tree accumulation potential and six agroclimatic factors using the Prediction Profiler. It was done by creating a matrix of plots with the X-axis representing the number of agroclimatic parameters and the Y-axis representing the AGB of coffee trees. The X-axis displays the values of agroclimatic variables, while the Y-axis desplays the values observed AGB. The mean and standard deviation in the normal distribution of parameters define the relationship between the AGB and indicators. At the average values of agroclimatic indicators such as 61.86 days of CDD, 46.55 days of R10 mm, 8.39 °C of TNn, 18.95 °C of TNx, and 18.74 °C of TXx, the mean value of AGB showed up at 26.66 kg/tree under the present climatic conditions (Figure 5a).

Under the RCP4.5 climate change scenarios, the average value of C. arabica's AGB would be 28.79 kg, with average values of 48.86 days for CDD, 45.47 days for R10mm, 7.78 °C for TNn, 17.78 °C for Nx, 19.92 °C for TXn, and 29.52 °C for TXx (Figure 5b). In the RCP8.5 scenarios, the average of *C. arabicatree's* AGB was also achieved at 24.41 kg when average values of all agroclimatic variables were measured concurrently at 66.19 days, 47.84 days, 8.53 °C, 18.21 °C, 18.72 °C, and 30.22 °C, respectively, for CDD, R10mm, TNn, TNx, TXn, and TXx (Figure 5c). It shows that the predicted AGB of the *C. arabica* tree under RCP4.5 will be higher than the predicted AGB under present and RCP48.5 climatic conditions. Despite this, the AGB anticipated by RCP8.5 is the smallest. If the predictors exceed or drop further beyond these limits, the AGB of coffee tree performance declines or rises depending on the explanator parametric estimation. TNn and TNx are the most critical factors in determining the AGB of *C. arabica’s* performance in current and future climatic conditions, respectively. It suggests that when the coldest minimum temperature increases in current climatic conditions, AGB decreases, and when the warmest minimum temperature increases in future climatic conditions, AGB will increase while the other variables stay constant.

### 3.4 Aboveground biomass estimation under climate change

#### 3.4.1 Descriptive statistics of the estimated coffee trees' AGB

Table 6 shows the descriptive statistics of the AGB of the *C. arabica* trees under three ecoclimatic regions and the entire study area. When the observed AGB dataset was subjected to an Artificial Neural Network (ANN) model, the mean values of predicted AGB in the current, RCP4.5, and RCP8.5 climatic conditions were found to be 26.66, 28.79, and 24.41 kg/tree, respectively. It means that the AGB of *C. arabica* tree was responsive to climate parameters, as it was elevated in coffee landscapes with favorable or green climate scenarios while decreasing in landscapes with adverse climatic circumstances.

**Table 6:** Descriptive statistics of predicted AGB of *C. arabica* tree under three microclimatic clusters.

Cluster	Decades	Scenarios	N	Mean (kg)	Std Dev	SE	CV	Min	Max	Range
1	Current	–	27	32.56	4.37	0.84	13.41	20.00	39.33	19.33
	2060s	RCP4.5	27	35.54	2.94	0.57	8.27	28.98	39.20	10.23
		RCP8.5	27	23.42	4.67	0.90	19.95	15.35	29.20	13.85
2	Current	–	49	29.92	2.39	0.34	8.00	26.09	36.02	9.93
	2060s	RCP4.5	49	28.23	5.69	0.80	20.16	17.08	36.54	19.46
		RCP8.5	49	24.50	5.10	0.72	20.83	16.19	33.42	17.23
3	Current	–	51	20.21	4.05	0.57	19.90	12.41	27.66	15.25
	2060s	RCP4.5	51	25.71	2.85	0.40	11.07	18.39	29.42	11.03
		RCP8.5	51	27.40	4.14	0.58	15.13	16.57	34.05	17.48
Overall	Current	–	127	26.66	6.34	0.56	23.79	12.41	39.33	26.92
	2060s	RCP4.5	127	28.79	5.56	0.49	19.32	17.08	39.20	22.12
		RCP8.5	127	25.41	4.90	0.43	19.26	15.35	34.05	18.70

Similar differences in AGB exist between ecoclimatic clusters as well. Under the current and RCP4.5 climate conditions, the predicted mean value of AGB in the first cluster was higher than the values in the second and third clusters. The expected mean value of AGB in the third cluster, on the other hand, was estimated to be higher than those values in the first and second clusters. This disparity might result from the maldistribution of the solar heating, land surface, the physical features of the areas, and their interconnections, all of which play a role in this variation.

#### 3.4.2 Aboveground biomass changes under different microclimates

Table 7 presents the changes in AGB of *C. arabica* trees under the current and future climate change scenarios via RCP4.5 and RCP8.5 in the 2060s. The result reveals that the performance of AGB accumulations in coffee trees in the three clusters was significantly different under the cyrrent, RCP4.5, and RCP8.5 climate change scenarios (P < 0.05).

**Table 7:** The mean difference between current and 2060s predicted A $\hat{G}$ B of *C. arabica* tree.

Cluster	Decades	Scenarios	N	Mean (kg)	A $\hat{G}$ B’s mean Change [compared to current]			
					Value (kg)	SE	%	P-value
1	Current	–	27	32.56	–	–	–	–
	2060s	RCP4.5	27	35.54	2.98	1.11	9.15	0.0232
		RCP8.5	27	23.42	‒9.14	1.11	‒28.07	<0.0001
2	Current	–	49	29.92	–	–	–	–
	2060s	RCP4.5	49	28.23	‒1.69	0.92	‒5.65	0.17
		RCP8.5	49	24.50	‒5.42	0.92	‒18.11	<0.0001
3	Current	–	51	20.21	–	–	–	–
	2060s	RCP4.5	51	25.71	5.50	0.74	27.21	<0.0001
		RCP8.5	51	27.40	7.19	0.74	35.58	<0.0001
Overall	Current	–	127	26.66	–	–	–	–
	2060s	RCP4.5	127	28.79	2.13	0.7	7.99	0.01
		RCP8.5	127	25.41	‒1.25	0.7	‒4.69	0.1814

*Note:*$A\hat{G}B$ indicates the predicted aboveground biomass in kilograms per tree, while (–) sign indicates a reduction in AGB as compared to the current climatic condition.

*In the first cluster*, the mean values of AGB under RCP4.5 were substantially higher than the mean value of AGB in the current ones (P = 0.023). Result indicates that, under the RCP4.5 scenario, the performance of AGB accumulation per coffee tree in the coming 2060s will be raised by 2.98 kg (9.15%) compared to current climatic circumstances. However, under the RCP8.5 scenarios, the AGB of coffee trees is expected to be substantially lower than it is now (P < 0.0001). The performance of AGB accumulation in the *C. arabica* tree will be 9.14 kg (28.07%) lower under the RCP8.5 scenarios. The *C. arabica* tree’s AGB accumulation will be higher in an intermediate carbon emission scenario than in the current one.

*In the second cluster,* the performance of AGB accumulation in *C. arabica* under RCP4.5 was projected to be worse in the 2060s than at its current stage. Result shows that under RCP4.5 circumstances, the performance of the *C. arabica* tree in terms of AGB accumulation will be insignificantly lower than it is now (P = 0.17). It would be lowered by 1.69 kg (5.65%) per tree. Furthermore, it would be significantly reduced under the RCP8.5 climatic scenario (P < 0.0001) from 28.23 to 24.50 kg/tree, or decreased by 5.42 kg (18.11%) in the 2060s. In conclusion, the AGB of the coffee tree would decrease under the medium carbon and highest carbon emission scenarios. However, the reduction would be more severe under the highest emission scenario than under the intermediate emission scenario over the second ecoclimatic cluster.

*In the third cluster,* this location was recognized as having the lowest coffee plant growth potential. However, the AGB of coffee plants was projected to positively respond to agroclimatic parameters in this ecological cluster (P < 0.0001). It will increase by 5.50 kg (27.21%) and 7.19 kg (35.58%) under the RCP4.5 and RCP8.5 scenarios, respectively, implying that the change is more robust in the highest emission scenario (RCP8.5) than those in the medium scenario (RCP4.5). It means that change in agroclimatic indicators has beneficial effects in this cluster.

*In the overall study area,* the productivity of the *C. arabica* tree was quantitatively altered as a result of changes in agroclimatic factors under the two distinct climate scenarios. The findings show that the mean value of AGB calculated under RCP4.5 was much greater than under the current conditions. The mean value of AGB under the RCP8.5 scenario, on the other hand, was expected to be much lower than the present one. It would rise considerably by 2.13 kg (7.99%) under RCP4.5 (P < 0.0001), but shrink significantly by 1.25 kg (4.69%) during RCP8.5 (P < 0.18).

## 4 Discussions

The productivity of *C. arabica* was predicted by this study using a GRM and ANNM for the current, RCP4.5, and RCP8.5 climate change scenarios in three different clusters. According to the predictive models, the AGB growth performance of the *C. arabica* tree and agroclimatic indicators are significantly correlated (P < 0.05). ​The increase in consecutive dry days (CDD) and warm night temperature (TNx) seemed to significantly reduce the *C. arabica* tree’s AGB under the current (the 2000s) and highest (RCP8.5) emission scenarios of the 2060s, whereas they seemed to significantly increase the plant growth parameter under the medium (RCP4.5) emission scenario by the 2060s. It follows that under the status quo, extreme agroclimatic indicators have a negative impact on the productivity of *C. arabica,* whereas they have a positive effect on it under some scenarios, with climate change mitigation and adaptation. The rise of the cold night (TNn), on the other hand, significantly improves the performance of the AGB of the *C. arabica* tree under the current climatic conditions, while it tends to degrade under the RCP8.5 scenarios. The productivity of the *C. arabica* species seems to benefit from the current rising cold nighttime temperatures. It is unexpected that under RCP8.5 emission scenarios, the AGB's accumulation performance of the *C. arabica* trees significantly increases under the rising extreme maximum temperature, while it decreases under the current and RCP4.5 climatic conditions. This might be related to the future increase of atmospheric Carbo dioxide and precipitation. The warmer temperatures with a higher level of atmospheric carbon dioxide and adequate water supplies may increase the productivity of crop plants (Moraes et al., 2010). However, under this emission scenario, the combined effects of agroclimatic indicators substantially reduce the performance of AGB coffee trees.

The performance of the *C. arabica* tree in terms of AGB accumulation might be related to variation in agroclimatic indicators in the three ecoclimatic clusters. The impact of agroclimatic change on *C. arabica*’s growth performance (AGB) in the study area in general and in the second cluster, in particular, will be much higher in the RCP4.5 scenario than in the RCP8.5 climate change scenario, as shown in Table 5. It suggests that coffee in different microclimates is more sensitive to an open-ended emission scenario in diverse ways. According to Pen et al. (2004), plant growth performance in a cold-wet environment is more susceptible to warming temperatures, while its growth performance in a warm-dry ecosystem is more susceptible to drought. The present findings are similar to those of Fu et al. (2017) who discovered a significant decrease in the AGB of larch plant species in China’s semi-arid climate zone as a result of extreme temperatures and abundant total precipitation in the wettest quarter, while excessive rainfall and high temperatures during the growing season may favor plant growth performance. Soil temperature extremes also affect the biomass of the plant, in which warmer soil temperatures in shallow layers increase the biomass allocation to above-ground plant parts, while the enhanced warming of frozen soil in deep layers caused by warming treatment produces more moisture that affects plant biomass allocation (Manhou et al., 2016).

*C. arabica* plants might perish due to unexpected climate change, especially when the flowers arrive early. The study by Drinnan and Menzel (1995) suggests that *C. arabica* growth performance slowed from January to May when daytime and nighttime temperatures climbed, then picked up again in the summer and autumn. According to Moat et al., (2017a, 2017b), plant AGB may acclimate to tight temperature variations of 18–22 °C. However, the total exposure of *C. arabica* to high temperatures may result in energy overpressure as it absorbs much more energy than that directed to photosynthesis (Moraes et al., 2010). On the other hand, warmer temperatures with a higher level of atmospheric carbon dioxide and adequate water supplies may reduce damage to *C. arabica* (Moraes et al., 2010). The present findings also support the study of Rodrigues et al. (2016), who found that lowering the minimum temperature disrupts plant nutrient intake.

When temperatures in the coldest season drop below 18 °C, it limits the economic performance of the crops (Bento et al., 2010). Since plants produce enzymes to break down the components around them, this process can be disrupted by freezing temperatures. They may have been stunted growth or, in the worst-case situation, the death of the plant as a result (Ramalho et al., 2014). The colder temperatures have a mild impact on coffee net photosynthesis, beginning around 20–18 °C and causing a severe and widespread impact when temperatures drop to 4 °C (Batista-Santos et al., 2011). In general, temperature and precipitation extreme indicators would wreak havoc on coffee’s development and reproductive phases (Hatfield and Prueger, 2015). The temperatures ranging from 18 to 25 °C and 5–10 mm of rainfall per day, on the other hand, are ideal for coffee development (Moat et al., 2017a, 2017b; van Oijen et al., 2010). Another work done by Fu et al. (2017) indicated that excessive rain during the growing season and a high mean temperature in the wettest quarter significantly reduced the AGB, while a warm growing season and abundant precipitation in the wettest quarter increased the AGB.

## 5 **C**onclusions

The increase in frequency and intensity of agroclimatic indicators has increased threats to Arabic coffee production. *C. arabica* grown in a separate eco-climatic region has been projected for AGB performance under current and future climate change scenarios. The technique is valuable for comprehending how climatic extremes influence the growing performance of coffee trees throughout diverse landscapes. In this study, agroclimatic extreme indicators have a two-fold effect on *C. arabica* AGB accumulation performance under the studied climate change scenarios, with some extremes assisting in increasing AGB while others hindering it. Because Arabica coffee has unique growth needs, even slight variations in temperature and precipitation have a significant impact on the plant growth performance.

The result of AGB in the second microclimatic cluster is expected to decrease. The increase in consecutive dry days (CDD) and rising night temperatures (TNx) now represent a greater danger to reduce coffee production than ever before in every setting of climate change scenarios. Smallholder farmers who cultivate coffee in areas with a higher prevalence of CDD and TNx will be unable to produce high-quality coffee and may be forced to abandon their planting. ​In contrast, AGB performance is estimated to be particularly higher in the third microclimatic cluster. Areas with a high frequency of heavy precipitation days (R10mm) and warmer daytime temperatures (TXx) considerably improve the performance of the coffee tree’s AGB in all climatic circumstances. It might be due to the combined effects of high amounts of CO2 in the atmosphere, warmer temperatures, and an abundance of soil moisture.

Therefore, incremental adaptation options such as crop replacement, new varieties, diversification, shade, and irrigation may be required for the second microclimatic cluster, and transformational adaptation options (e.g., expansion of coffee growing in new areas) may be required for the third microclimatic cluster. In addition, more studies into the impacts of other climatic factors, such as biologically effective degree days, cold, hot, and wet spells on crops in different localities will be needed.

## Declarations

### Author contribution statement

Fedhasa Benti Chalchisa: Conceived and designed the experiments; Performed the experiments; Analyzed and interpreted the data; Contributed reagents, materials, analysis tools or data; Wrote the paper.

Girma Mamo Diga, Gudina Legese Feyisa, and Alemayehu Regassa Tolossa: Conceived and designed the experiments; Analyzed and interpreted the data; Contributed reagents, materials, analysis tools, or data.

### Funding statement

This work was supported by Jimma University, non-profit making Orgainization.

### Data availability statement

The data that has been used is confidential.

### Declaration of interest’s statement

The authors declare no conflict of interest.

### Additional information

No additional information is available for this paper.
